# A “resistance calculator”: Simple stewardship intervention for refining empiric practices of antimicrobials in acute-care hospitals

**DOI:** 10.1017/ice.2020.1372

**Published:** 2021-09

**Authors:** Shani Zilberman-Itskovich, Nathan Strul, Khalil Chedid, Emily T. Martin, Akram Shorbaje, Itzhak Vitkon-Barkay, Gil Marcus, Leah Michaeli, Mor Broide, Matar Yekutiel, Yarden Zohar, Hadas Razin, Amitai Low, Ariela Strulovici, Boaz Israeli, Gal Geva, David E. Katz, Eli Ben-Chetrit, Mutaz Dodin, Sorabh Dhar, Leo Milton Parsons, Abdiel Ramos-Mercado, Keith S. Kaye, Dror Marchaim

**Affiliations:** 1 Shamir (Assaf Harofeh) Medical Center, Zerifin, Israel; 2 University of Michigan School of Public Health, Ann Arbor, Michigan, United States; 3 Sackler School of Medicine, Tel-Aviv University, Tel-Aviv, Israel; 4 Shaare Zedek Medical Center, the Hebrew University Hadassah Medical School, Jerusalem, Israel; 5 Detroit Medical Center, Detroit, Michigan, United States; 6 Wayne State University School of Medicine, Detroit, Michigan, United States; 7 University of Michigan Medical School, Ann Arbor, Michigan, United States

## Abstract

**Objective:**

In the era of widespread resistance, there are 2 time points at which most empiric prescription errors occur among hospitalized adults: (1) upon admission (UA) when treating patients at risk of multidrug-resistant organisms (MDROs) and (2) during hospitalization, when treating patients at risk of extensively drug-resistant organisms (XDROs). These errors adversely influence patient outcomes and the hospital’s ecology.

**Design and setting:**

Retrospective cohort study, Shamir Medical Center, Israel, 2016.

**Patients:**

Adult patients (aged >18 years) hospitalized with sepsis.

**Methods:**

Logistic regressions were used to develop predictive models for (1) MDRO UA and (2) nosocomial XDRO. Their performances on the derivation data sets, and on 7 other validation data sets, were assessed using the area under the receiver operating characteristic curve (ROC AUC).

**Results:**

In total, 4,114 patients were included: 2,472 patients with sepsis UA and 1,642 with nosocomial sepsis. The MDRO UA score included 10 parameters, and with a cutoff of ≥22 points, it had an ROC AUC of 0.85. The nosocomial XDRO score included 7 parameters, and with a cutoff of ≥36 points, it had an ROC AUC of 0.87. The range of ROC AUCs for the validation data sets was 0.7–0.88 for the MDRO UA score and was 0.66–0.75 for nosocomial XDRO score. We created a free web calculator (https://assafharofe.azurewebsites.net).

**Conclusions:**

A simple electronic calculator could aid with empiric prescription during an encounter with a septic patient. Future implementation studies are needed to evaluate its utility in improving patient outcomes and in reducing overall resistances.

The burden associated with common human pathogens becoming resistant to the majority of prescribed antimicrobials has been defined by the World Health Organization (WHO) as one of the current greatest threats to humanity.^1^ Multidrug-resistant organisms (MDROs) and extensively drug-resistant organisms (XDROs),^2^ are prevalent in hospitals, in non–acute-care settings, and even in the community.^3^ Infections with MDROs and XDROs result in significantly worse outcomes, partially due to delay in initiation of appropriate antimicrobial therapy (DAAT).^4^ In severe sepsis, DAAT is the strongest modifiable independent predictor for mortality.^4^ However, the widespread use of empiric, broad-spectrum therapy by providers further increases the burden of resistance.^5^ Moreover, when the infection results from a susceptible strain, some of the broad-spectrum agents (eg, vancomycin, colistin), are less efficacious and more toxic than narrower-spectrum agents.^6^ This “double-edged sword” situation is harmful both to individual patients and to the ecology in general.^7^

Previous studies have used various definitions for healthcare-associated infection in an attempt to predict resistance phenotypes.^8^ These definitions have also been incorporated into official treatment guidelines,^9^ such as the Duke-2002 criteria^8^ or the modified Duke-2002 criteria, to guide the empiric initiation of broad-spectrum agents.^10^ Further work has shown that these definitions perform poorly in predicting resistance.^11^ Some prescribers use scores composed of acute illness indices, such as the APACHE-II for septic shock^12^ or the CURB-65 for community-onset pneumonia,^13^ to guide empiric coverage for MDROs, even though those scores were developed and validated for other purposes. Therefore, prescribers need directive and reliable tools to improve appropriate targeting of antimicrobials to populations at high risk of MDROs or XDROs and to reduce the misuse of broad-spectrum therapeutics.^7^

The management at the initial acute phase of sepsis is crucial to patient outcomes.^14^ There are 2 common time points at which decisions are made pertaining to initial management of acute sepsis, and errors frequently occur.^7^ The first is upon admission (UA), with regard to MDROs such as extended-spectrum β-lactamase–producing Enterobacterales (ESBL),^15^ methicillin-resistant *Staphylococcus aureus* (MRSA),^16^ and *Pseudomonas aeruginosa*. The second is in cases of nosocomial infection, with regard to XDROs, such as vancomycin-resistant *Enterococcus* (VRE),^17^ and carbapenem-nonsusceptible gram-negative bacteria, such as Enterobacterales (CRE),^18^ *Acinetobacter baumannii* (CRAB),^19^ and *P. aeruginosa* (CRPA).^20^

Prior attempts to develop tools to predict resistance have been limited by several factors. Some scores lacked comprehensiveness and were either focused on a specific pathogen (eg, differentiating ESBL from CRE),^21^ on a specific drug (eg, predicting resistances to piperacillin-tazobactam, cefepime, or meropenem in the ICU),^22^ or were limited to patients with a single infectious syndrome (eg, pneumonia or UTI).^23,24^ These studies addressed certain aspects of empiric prescription, but they did not address the whole process of decision making that prescribers face, particularly among cognitively impaired patients, in which the infectious syndrome (and naturally the offending pathogen) at the initial acute phase are difficult to determine.^7^ Moreover, prior scores were limited by low performance,^22^ small sample size,^25^ the absence of validation data sets,^22^ requiring parameters that were unpractical to extract bedside (eg, Charlson scores),^25,26^ or reliance on additional non–standard-of-care testing (eg, arterial blood gases to calculate APACHE for patients with mild sepsis).^22^ In addition, prescribers in general are reluctant to memorize scores.^7^

We aimed to generate a simple electronic calculator to predict both MDRO UA and nosocomial XDRO infections, and only require parameters to be entered which are readily available at the bedside.

## Methods

We conducted a retrospective cohort study at Shamir Medical Center (SMC), Israel. The derivation data set for the MDRO UA score consisted of patients with sepsis admitted to the emergency room from August 28, 2016, to December 12, 2016 (overall, 106 consecutive days, a convenience sample based on a-priori power calculations). The derivation data set for the nosocomial XDRO score consisted of hospitalized septic patients from the entire calendar year of 2016. The institution’s ethics committee approved the study prior to initiation.

We applied the following inclusion criteria: patients aged >18 years, with presence of sepsis,^27^ from whom blood cultures were obtained. According to hospital regulations, blood cultures are ordered from every hospitalized patient with sepsis, disregarding its severity or its origin. Data used for the derivation of the MDRO UA score data set were restricted to patients with sepsis from whom blood cultures were obtained during the first 2 calendar days of hospitalization (ie, the day of ER admission was considered day 1). The nosocomial XDRO score was based on a data set of septic patients from whom blood cultures were obtained on day 4 of hospitalization and thereafter. Patients from whom blood cultures were drawn on day 3 of hospitalization were not included in either data set. Patients with monomicrobial, microbiologically confirmed, anaerobic or fungal infection were excluded because these infections are subjected to a different set of prediction rules.^28^

### Multidrug-resistant organisms (MDROs) and extensively drug-resistant organisms (XDROs)

We utilized broad definitions of MDROs and XDROs as determined by an international consortium in 2012,^2^ and we modified them to include pathogens resistant to the initial first line of therapy recommended by official guidelines.^29^ MDROs were defined as follows: (1) MRSA, (2) ampicillin-nonsusceptible enterococci, (3) penicillin- or ceftriaxone-nonsusceptible *Streptococcus pneumoniae*, (4) *P. aeruginosa*, (5) *A. baumannii*, or (6) Enterobacterales nonsusceptible to 1 or more third-generation cephalosporin (eg, ceftriaxone, ceftazidime).

XDROs were defined as follows: (1) VRE, (2) heterogeneous vancomycin-intermediate *S. aureus* (hVISA) or *S. aureus* with MIC≥2 to vancomycin,^30^ (3) CRE (Enterobacterales with evidence of carbapenemase production and/or with meropenem MIC≥2),^31^ (4) CRAB, (5) CRPA, and (6) intrinsically carbapenem-nonsusceptible gram-negative bacteria (eg, *Stenotrophomonas maltophilia* and *Burkholderia cepacia*).

### Data collection derivation data set

Clinical data were retrieved from all records. Mortality data were retrieved from a national registry governed by the Israeli Ministry of the Interior. More than 200 potential score components were captured (Supplementary Table 1 online), including demographics, healthcare exposures, comorbidities, acute illness indices, microbiological data, antimicrobial treatments, and outcomes. The infectious clinical syndrome was determined based on established criteria.^32^ The microbiological diagnosis was determined based on blood culture results or culture results from nonsterile sites that were coupled to the appropriate infectious syndrome (eg, respiratory culture for patients with pneumonia, urine culture for patients with urinary-tract infection and so on). Only parameters that were readily available at the bedside to a clinician equipped with an electronic chart were incorporated into the models.

**Table 1.** Univariable Analyses of Features Associated With Septic Patients with Multidrug-Resistant Organism (MDRO) Infection Upon Admission (UA) Versus Those With Non-MDRO UA infection, and of Patients With Nosocomial Extensively Drug-Resistant Organism (XDRO) Infection Versus Patients With Non-XDRO Nosocomial Infection (Derivation Data Sets)

Parameter	MDRO		Non-MDRO		MDRO vs Non-MDRO		XDRO		Non-XDRO			XDRO vs Non-XDRO
	No.	%	No.	%	OR (CI-95%)	*P* Value	No.	%	No.	%	OR (CI-95%)	*P* Value
Population	165		1371				167		635			
**Demographics**												
Age, mean y ± SD	76 ± 15		60 ± 22			<.001	76 (18–103)		77 (18–103)			.566
Elderly (aged >65 y)	139	84.2	688	50.2	5.3 (3.4–8.2)	<.001	132	79.5	486	76.4	1.2 (0.8–1.8)	.40
Sex, male	81	49.1	586	42.7	1.3 (0.9–1.8)	.12	97	58.4	338	53.1	1.24 (0.9–1.7)	.22
**Exposure to healthcare environments or procedures**												
Recent (<3 mo) hospitalization	75	45.5	324	23.6	2.8 (2.0–3.9)	<.001	65	39.2	248	39	1 (0.7–1.4)	.97
LTCF resident	46	27.9	139	10.1	3.4 (2.3–5.0)	<.001	48	28.9	103	16.2	2.1 (1.4–3.1)	<.001
Recent (<3 mo) LTCF stay prior to hospitalizing	41	24.8	101	8.8	3.4 (2.3–5.1)	<.001						
Advanced nursing or intravenous therapy at home	11	6.7	12	0.9	8.1 (3.5–18.6)	<.001						
Regular visits (at least weekly) to outpatient clinics	15	9.1	64	4.7	2.0 (1.1–3.7)	.015						
Hemodialysis	5	3	36	2.6	1.2 (0.4–3.0)	.76	6	3.6	37	5.8	0.6 (0.3–1.5)	.25
Any antibiotic course in the preceding 3 months	108	65.5	439	37.3	3.9 (2.7–5.5)	<.001	163	98.2	448	70.4	22.8 (7.2–72.2)	<.001
Invasive procedure^1^ in the preceding 6 mo	51	30.9	183	13.3	2.9 (2.0–4.2)	<.001	84	50.6	296	46.5	1.2 (0.8–1.7)	.351
Permanent device^2^	37	22.4	136	9.9	2.6 (1.7–3.9)	<.001	47	28.3	140	22	1.4 (1–2.1)	.087
ICU stay in preceding 3 mo	5	3	8	0.6	5.3 (1.7–16.5)	.001	15	22.7	8	3.6	7.9 (3.2–19.6)	<.001
ICU (or advanced-care room) stay in current hospitalization							140	83.8	179	28.1	13.2 (8–20.6)	<0.001
MDRO carrier^3^ from the past 2 y	69	41.8	114	8.3	7.9 (5.5–11.4)	p<0.001	112	67.5	153	24.1	6.6 (4.5–9.5)	<0.001
**Background medical status and conditions**												
Functionally dependent	112	67.9	428	31.2	4.7 (3.3–6.6)	<.001	132	79.5	256	40.3	5.8 (3.8–8.7)	<.001
Altered consciousness/cognitive impairment	63	38.2	213	15.5	3.4 (2.4–4.7)	<.001						
Ischemic heart disease	47	28.5	276	20.1	1.6 (1.1–2.3)	.13	60	36.1	228	35.8	1 (0.7–1.4)	.944
Congestive heart failure	36	21.8	172	12.5	1.9 (1.3–2.9)	.001	67	40.4	251	39.5	1 (0.7–1.5)	.834
Diabetes mellitus	74	44.8	412	30.1	1.9 (1.4–2.6)	<.001	75	45.2	285	44.8	1 (0.7–1.4)	.932
Chronic kidney disease	43	26.1	206	15	2.0 (1.4–2.9)	<.001	38	22.9	167	26.3	0.8 (0.6–1.2)	.376
Chronic lung disease	36	21.8	241	17.6	1.3 (0.9–1.9)	.18	85	51.2	212	33.3	2 (1.5–3)	<.001
Hemi/para plegia/paresis	17	10.3	44	3.2	3.5 (1.9–6.2)	<.001	102	61.4	254	39.9	2.4 (1.7–3.4)	<.001
Dementia	59	35.8	203	14.8	3.2 (2.3–4.6)	<.001	75	45.2	176	27.7	2.2 (1.5–3.1)	<.001
Active malignancy	24	14.5	118	8.6	1.8 (1.1–2.9)	.013	24	14.5	140	22	0.6 (0.4–0.96)	.032
Immunosuppression^4^	31	18.8	189	13.8	1.5 (0.95–2.2)	.083	66	39.8	171	26.9	1.8 (1.3–2.6)	.001
Charlson score, mean ± SD												
Weighted index comorbidity	3 ± 2		2 ± 2			<.001	5.8 ± 2.9		5.1 ± 3.1			.004
Combined condition score	6 ± 3		4 ± 3			<.001	8.7 ± 3.4		7.8 ± 3.9			.004
10-y survival, %	11 ± 7		7 ± 7			<.001	1 (0–98)		1 (0–98)			.003
**Acute illness indices**												
Severe sepsis, septic shock, multiorgan failure (previous sepsis severity classification)	96	58.2	348	25.4	4.1 (2.9–5.7)	<.001	118	71.1	201	31.6	5.3 (3.7–7.7)	<.001
ICU at culture date	28	17	93	6.8	2.8 (1.8–4.4)	<.001	140	83.8	179	28.1	13.2 (8–20.6)	<.001
Ventilated at culture date	26	15.8	93	6.8	2.6 (1.6–4.1)	<.001	32	57.1	75	13.8	8.4 (4. 7–15)	<.001
Acute kidney injury	73	45.9	256	19.3	3.6 (2.5–5.0)	<.001	73	45.6	149	24.8	2.5 (1.8–3.6)	<.001
Altered consciousness/cognition	83	50.3	297	21.7	3.7 (2.6–5.1)	<.001	141	84.9	297	46.7	6.4 (4.1–10.1)	<.001
Chronic ulcer	32	19.4	52	3.8	6.1 (3.8–9.8)	<.001	61	36.7	151	23.7	1.9 (1.3–2.7)	.001
Rapidly fatal McCabe score	42	25.5	125	9.1	3.4 (2.3–5.1)	<.001	104	62.7	174	27.4	4.5 (3.1–6.4)	<.001
Pitt score, median (IQR)	1 (0–10)		1 (0–1)			<.001	6.7 (0–14)		2.7 (1–10)			<.001
**Clinical syndrome**												
Urinary tract	89	53.9	285	20.8		<.001	12	7.2	139	21.9	0.3 (0.2–0.5)	<.001
Respiratory	38	23	514	37.5		<.001	115	69.3	228	35.8	4 (2.8–5.7)	<.001
Skin or soft tissue	29	17.6	188	13.7	1.3 (0.9–2.1)	.18	12	7.2	80	12.6	0.5 (0.3–1)	.05
Intra-abdominal	4	2.4	181	13.2	0.16 (0.06–0.45)	<.001	2	1.2	48	7.5	0.15 (0.04–0.6)	.003
Endocarditis	0	0	11	0.8		.6	0	0	3	0.5		.37
Primary bloodstream infection	0	0	17	1.2		.24	22	13.3	74	11.6	1.2 (0.7–1.9)	.57
Central nervous system	0	0	22	1.6		.16	1	0.6	1	0.2		.30
Bacteremia without determined focus	4	2.4	35	2.6	0.95 (0.33–2.7)	>.999	2	1.2	62	9.7		<.001
Gynecologic or pelvic	1	0.6	118	8.6	0.06 (0.01–0.47)	<.001	0	0	1	0.2		>.99
**Hospitalization division**												
Medicine (including advanced-care rooms)	103	62.4	873	63.7	0.95 (0.7–1.3)	.75	19	11	308	48.5	0.13 (0.1–0.2)	<.001
Surgery	18	10.9	139	10.1	1.1 (0.6–1.8)	.76	8	5	140	22	0.2 (0.1–0.4)	<.001
Obstetrics gynecology	3	1.8	198	14.4	0.1 (0.03–0.35)	<.001	0	0	8	1.3		.22
ICU or ICCU	33	20	99	7.2	3.2 (2.1–5.0)	<.001	140	84	179	28	13.2 (8–20.6)	<.001
**Antimicrobial therapy**												
Days to appropriate therapy, median (range)	1 (0–3)		0 (0–1)			<.001	2 (0–12)		1 (0–8)			<.001
≥48 h delay in initiating appropriate therapy	100	60.6	30	9.2	15.2 (9.3–25)	<.001	90	64	99	36.7	3 (2–4.65)	<.001
**Outcomes**												
LOS from infection to discharge after excluding the dead, median d (range)	8 (5–12)		5 (3–8)			<.001	52.5 (6–251)		20 (5–669)			<.001
Died in current hospitalization	39	23.6	103	7.5	3.8 (2.5–5.8)	<.001	109	65.7	178	28	4.9 (3.4–7.1)	<.001
Died within 14 d	29	17.9	76	5.7	3.6 (2.3–5.8)	<.001	78	47	145	22.8	3 (2.1–4.3)	<.001
Died within 90 d	54	37	163	11.9	3.6 (2.5–5.3)	<.001	113	68.1	250	39.3	3.3 (2.3–4.7)	<.001
**Among survivors of the index hospitalization only**												
Functional status deterioration at discharge	23	18.3	117	9.2	2.2 (1.3–3.6)	.001	41	73.2	214	47.1	0.7 (0.5–0.96)	.001
Discharge to LTCF (only patients admitted from home)	7	6.6	53	4.5	1.5 (0.7–3.4)	.32	28	50	148	32.7	3.1 (1.6–5.8)	<.001
Additional hospitalization in the following 3 mo	62	54.4	286	27	3.2 (2.2–4.8)	<.001	22	44	136	34.5	0.6 (0.3–0.9)	.02

Note. MDRO, multidrug-resistant organism; OR, odds ratio; CI, confidence interval; LTFC, long-term care facility; S/P, stats post; CABG, coronary artery bypass graft; CVA, cerebrovascular accident; TIA,- transient ischemic attack; AIDS, acquired immune deficiency syndrome; SIRS, systemic inflammatory response syndrome; ICU, intensive care unit ICCU, intensive cardiac care unit; LOS, length of stay; SD, standard deviation; IQR, interquartile range; LOS, length of stay.

### Validation data sets

The score performances were tested on different historical validation data sets. Among them, 6 were from SMC and 1 was from Detroit Medical Center. The same inclusion, exclusion, and stratification criteria were applied to all data sets. In a few of the validation data sets, only patients with positive blood culture results were included. The MDRO UA score was validated for 3 data sets, and the nosocomial XDRO score was validated for 4 data sets (Supplementary Table 2 online).

**Table 2.** Multivariable Model of Risk Factors for MDRO^a^ Upon Admission (Derivation Data Set)

Variable	Score	Odds Ratio	*P* Value	95% Confidence Interval
Regular visits (at least weekly) to outpatient clinic, including hemodialysis	17	5.31	<.001	2.28–12.37
MDRO^a^ carrier from the preceding 2 y	14	4.09	<.001	2.65–6.32
Advance nursing or intravenous therapy delivered at home	12	3.45	.027	1.15–10.35
Elderly (aged >65 y)	12	3.34	<.001	2.65–6.32
Antibiotic treatment (≥2 d duration) in the past 3 mo	9	2.44	<.001	1.62–3.67
Invasive procedure^b^ in the preceding 6 mo	9	2.44	<.001	1.53–3.87
Hemi/para plegia/paresis^c^	7	2.04	.052	0.995–4.18
Severe sepsis, septic shock, multiorgan failure (previous sepsis severity classification)	5	1.68	<.001	1.02–2.77
Altered consciousness in background^d^	5	1.61	.047	1.01–2.57
Acute kidney injury^e^	4	1.57	.056	0.99–2.49

Note. MDRO, multidrug-resistant organism.

a MDRO include (1) Methicillin-resistant *staphylococcus aureus* (MRSA), (2) ampicillin non-susceptible enterococci, (3) penicillin or ceftriaxone nonsusceptible *Streptococcus pneumoniae*, (4) *Pseudomonas aeruginosa*, (5) *Acinetobacter baumannii*, and (6) Enterobacterales nonsusceptible to 1 or more third-generation cephalosporin (eg, ceftriaxone, ceftazidime).

b This includes any type of invasive procedure, such as surgery, endoscopy, central-line placement, any percutaneous procedure (eg, coronary angiography and paracentesis).

c This refers to the status of the patient prior to infection onset (include bed-ridden).

d This includes dementia, disorientation, confusion, or coma.

e An acute rise in serum creatinine level: ie, >0.3 mg/dL, by >50% of baseline, or a decrease in urine output for >6 h.

### Statistical analyses

All analyses were performed using Stata version 13.1 software (StataCorp, College Station, TX). Regarding model validation, all variables of interest were tested using a forward-stepwise logistic regression model requiring a *P* value <.15 by the likelihood ratio test for inclusion. After the addition of significant variables, variables with a *P* value ≥.20 were excluded. Variables with a negative coefficient in the model were then excluded for the utility of the prediction score. Each remaining parameter was assigned a score equal to 10 times its coefficient, and scores for individual parameters were summed to determine a total score. A receiver operating characteristic (ROC) curve was generated and the area under the curve (AUC) was calculated to assess model performance. Sensitivity, specificity, positive predictive values (PPV) and negative predictive value (NPV) were calculated following the assignment of a cutoff score. A free web-based calculator was created that responds to all platforms of stationary and mobile devices.

## Results

Overall, 4,114 patients were included: 2,472 patients in the derivation (n = 1,536) and validation (n = 936) data sets of the MDRO UA score and 1,642 patients in the derivation (n = 802) and validation (n = 840) data sets of the nosocomial XDRO score.

### Descriptive characteristics of individuals in the derivation data sets

We included 1,536 consecutive adults with sepsis UA and 802 with nosocomial sepsis. Most patients were elderly (53.8% of the UA data set and 77.1% of the nosocomial data set), with a mean and median age of 62 ± 22 years for the UA data set and 77 years (range, 18–103) for the nosocomial data set. Individuals in both data sets had frequent interaction with healthcare settings and invasive procedures prior to the index hospitalization, including permanent residency at long-term care facilities (11% of the UA data set and 19% of the nosocomial data set), recent (<6 months) invasive procedure (15% of the UA data set and 47% of the nosocomial data set), and previous recent (<3 month) hospitalizations (25% of the UA data set and 39% of the nosocomial data set). The mean Charlson combined condition scores were 4.2 ± 3.5 for the UA data set and 8 ± 3.8 for the nosocomial data set.^26^ Past MDRO carriage was evident among 12% of the UA data set and 33% of the nosocomial data set, and previous recent (<3 months) antibiotic therapy was documented among 41% of the UA data set and 76% of the nosocomial data set. Respiratory infections were the most common infectious syndrome among patients with sepsis UA (36%) and among patients with nosocomial sepsis (43%). The complete descriptive analysis of both populations is provided in Supplementary Table 1 (online).

### Microbiological distribution of offending pathogens (derivation data sets)

Definitive microbiological diagnosis of the causative offending pathogen(s) was achieved in 33% (501 of 1,536) of patients with sepsis UA (19% of them had bacteremia) and among 56% (448 of 802) of patients with nosocomial sepsis (20% had bacteremia). Of the pathogens associated with sepsis UA, 165 (11%) were MDROs, most commonly ESBLs (6%), *P. aeruginosa* (3%), and MRSA (1%). Of patients with nosocomial sepsis, 21% were XDROs, mainly CRAB (12.5%), CRPA (2.4%), and CRE (2.4%). The complete microbiological descriptive data are provided in Supplementary Table 3 (online).

### The MDRO upon admission score

Patients with MDRO infection were significantly older and had higher rates of recent exposure to healthcare and to antibiotics (Table 1). Multiple background conditions were associated with MDROs, including impaired functional status and cognition at baseline as well as enhanced acute illness indices. As expected, DAAT was more common in patients with MDROs, and clinical outcomes were worse (Table 1).

**Fig. 1.**
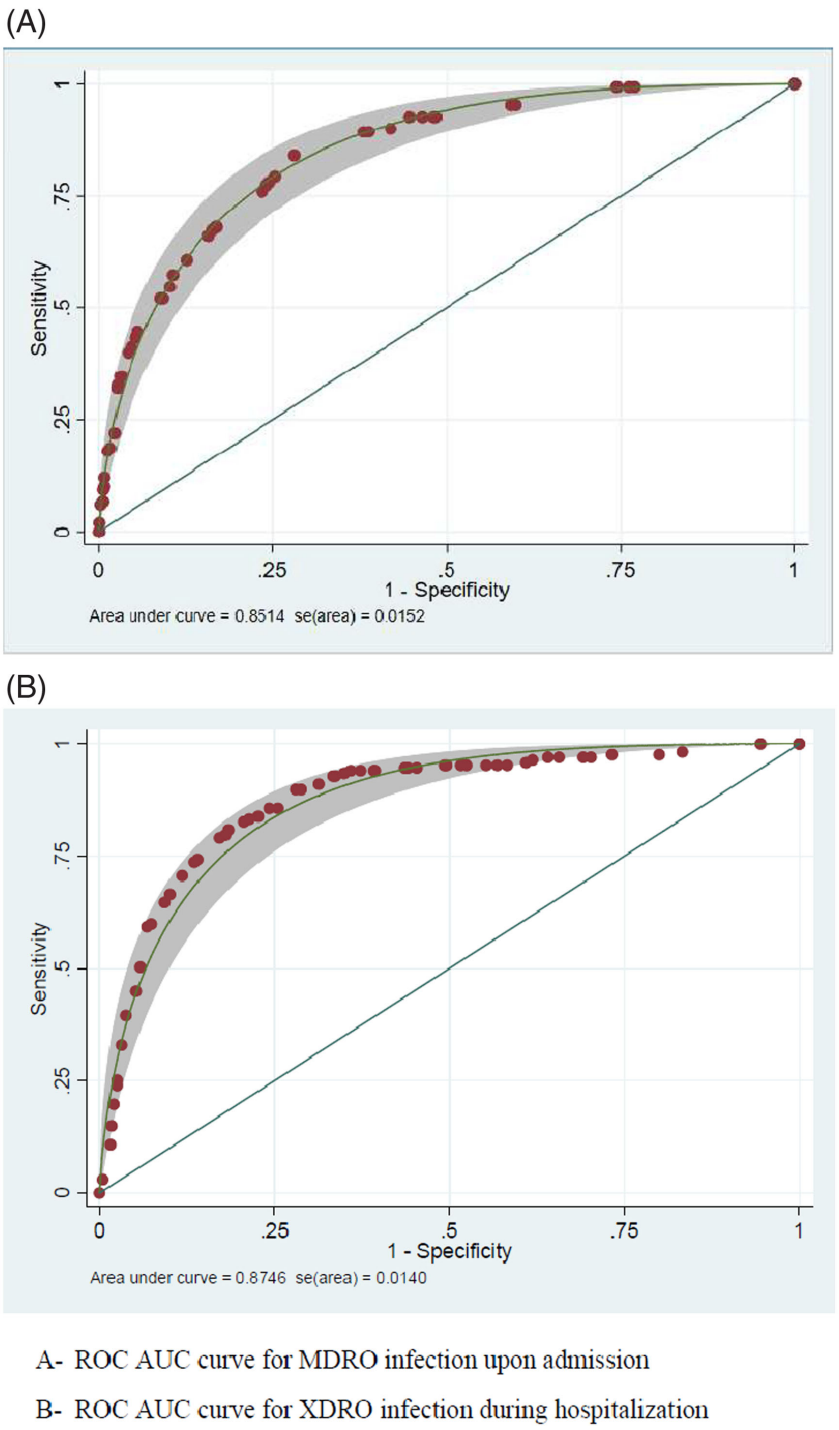
ROC AUC curve for MDRO-scoring system and XDRO-scoring system. (A) ROC AUC curve for MDRO infection upon admission. (B) ROC AUC curve for XDRO infection during hospitalization. Note. ROC AUC, area under the receiver operating characteristic curve; MDRO, multidrug-resistant organisms; XDRO, extensively drug-resistant organisms.

Multivariable analysis of risk factors was performed, and the final score components are depicted in Table 2. A cutoff of ≥22 points yielded 86% sensitivity, 67% specificity, 24% PPV, 98% NPV, and an ROC AUC of 0.85 (95% confidence interval [CI], 0.82–0.88) (Fig. 1). The application of the Duke-2002 criteria^8^ to this data set yielded an ROC AUC of 0.62 (70% sensitivity, 61% specificity, 18% PPV, 94% NPV), and the modified Duke-2002 criteria^10^ yielded an ROC AUC of 0.69 (68% sensitivity; 61% specificity; 17% PPV; 94% NPV). The APACHE-II^12^ and the CURB-65 (only among patients with pneumonia)^13^ yielded ROC AUCs of 0.8 and 0.75, respectively.

### The nosocomial XDRO score

Among patients with nosocomial sepsis, prior exposures to healthcare settings were much more substantial among patients with XDROs including recent long-term care facility (LTCF) stays, exposure to antibiotics, or past carriage of MDRO/XDRO. Indexes of comorbidities^26^ and acute illness indices (ie, severity of sepsis,^27^ Pitt bacteremia score,^33^ and McCabe score^34^) were also elevated among XDRO patients. Patients with nosocomial XDRO infections had frequent DAAT, and in nearly half of these patients, appropriate initiation of therapy was delayed for >2 days. This delay was reflected in significantly worse outcomes (Table 1). The final nosocomial XDRO score included 7 parameters as depicted in Table 3. Using 36 points as the cutoff yielded 90% sensitivity, 72% specificity, 46% PPV, 97% NPV, and an ROC AUC of 0.87 (95% CI, 0.84–0.91) (Fig. 1).

**Table 3.** Multivariable Model of Risk Factors for Nosocomial XDRO Infection (derivation data set)

Variable	Score	Odds Ratio	*P* Value	95% Confidence Interval
In ICU (or in advanced-care room) at infection onset	20	7.41	<.001	4.14–13.27
Known MDRO^a^ carriage from the previous 2 y	15	4.58	<.001	2.96–7.09
Pneumonia	11	3.05	<.001	1.96–4.77
Severe sepsis, septic shock, multiorgan failure (previous sepsis severity classification)	10	2.67	<.001	1.72–4.16
Hemi/para plegia/paresis^b^	5	1.64	.029	1.05–2.55
Invasive procedure^c^ in the preceding 6 mo	3	1.36	.163	0.88–2.10
Sex, male	3	1.29	.248	0.84–1.99

Note. XDRO, extensively drug-resistant organism; ICU, intensive-care unit; MDRO, multidrug-resistant organism.

a MDRO include (1) Methicillin-resistant *staphylococcus aureus* (MRSA), (2) ampicillin non-susceptible enterococci, (3) penicillin or ceftriaxone nonsusceptible *Streptococcus pneumoniae*, (4) *Pseudomonas aeruginosa*, (5) *Acinetobacter baumannii*, and (6) Enterobacterales nonsusceptible to 1 or more third-generation cephalosporin (eg, ceftriaxone, ceftazidime).

b This refers to the status of the patient prior to infection onset (include bed-ridden).

c This refers to any type of invasive procedure, including any type of surgery (from minor to major, ie, the whole spectrum), endoscopies, permanent central line insertions, any percutaneous procedure (eg, coronary angiography), ascites paracentesis, and more.

### The validation data sets

Table 4 reflects the performances of the 7 different validation historical data sets. The ROC AUC range was 0.7–0.88 among the validation data sets of the MDRO UA score, and the ROC AUC range was 0.66–0.75 among the validation data sets of the nosocomial XDRO score.

**Table 4.** Score Performance Characteristics (Validation Data Sets)

Validation Data Sets of the MDRO Upon Admission Score								Validation Data Sets of the Nosocomial XDRO Score							
Data Set Name	PI	Patient No.	Sensitivity, %	Specificity, %	PPV, %	NPV, %	ROC AUC	Data Set Name	PI	Patient No.	Sensitivity, %	Specificity, %	PPV, %	NPV, %	ROC AUC
SMC MDRO1	Gil Marcus	426	84	56	47	89	0.75	DMC CRE1	Violet Libman	182	63	68	16	95	0.71
SMC MDRO2	Mor Broide	199	89	71	52	94	0.88	SMC XDRO1	Matar Yekutiel	257	59	79	25	94	0.75
SMC MDRO3	Eyal Taleb	311	79	49	43	83	0.70	SMC XDRO2	Dana Leveat	162	65	78	46	88	0.74
								SMC XDRO3	Itzhak Vitkon-Barkay	239	76	51	68	61	0.66

PI, primary investigator; MDRO, multidrug-resistant organism; XDRO, extensively drug-resistant organism; ROC-AUC, the area under the receiver operating characteristic curve; SMC, Shamir Medical Center; PPV, positive predictive value; NPV, negative predictive value.

### Web calculator

We created a free web calculator (https://assafharofe.azurewebsites.net).

## Discussion

In this study, we present a simple, bedside tool to assist in empiric prescription of antibiotics during the first encounter with a septic patient. This tool could be implemented as a stewardship intervention in hospitals. For the past 80 years, antibiotics have been an efficacious and safe therapeutics, but there are concerns of it becoming a ‘wasted resource,’^35^ due to frequent errors in empiric prescription of broad-spectrum agents.^36^ Practitioners need reliable tools to guide empiric decisions and to avoid errors, which can be harmful to patients and to facilities. To our knowledge, this tool is among the largest score creation efforts for MDROs and XDROs conducted to date. In comparison with previous studies,^21,22,24^ the performance of our calculator was better. Moreover, this calculator addresses a wider range of situations, reflecting the broader perspective anticipated from a clinician, while managing a patient with acute sepsis. The high NPVs in both scores, of 98% and 96%, could assist prescribers in safely avoiding overuse of broad-spectrum agents. This tool could be a simple and effective stewardship intervention; broad-spectrum agents, on top of their detrimental ecological impact and the fiscal burden they impose, are also frequently less efficacious and more toxic to the patient.^6^ Prescribers are already using “prediction tools” that are less established were not originally developed to predict resistances and displayed much lower performances (both in the original studies and herein): the Duke-2002,^8^ modified Duke-2002,^10^ APACHE-II,^12^ and CURB-65 (for patients with community-onset pneumonia).^13^

Mobile devices are used nowadays by nearly every prescriber worldwide, including in developing countries. The calculator is responsive with all devices and is compatible with network security settings. A shortcut to this calculator could be pasted to home screens of private devices, and to organizational computers located at ERs, regular floors, and in ICUs. The calculator is anonymous; it is designed to assist hospitalists, internists, surgeons, intensivists, clinical pharmacists, and every adult prescriber. However, the calculator should not be used in lieu of a comprehensive medical evaluation, and it should not replace infectious diseases (ID) consultation when appropriate. ID experts will always be needed to tailor management, and in many facilities, they are responsible for prescription of broad-spectrum antibiotics. Therefore, the calculator may be able also to assist prescribers in determining when a formal ID consultation is needed. Sometimes a unique clinical or epidemiological finding is evident: a pathognomonic rash, travel to a certain area, acknowledgement of current epidemics. These unique scenarios necessitate special management considerations and usually the direct involvement of an ID specialist. When no unique exposure or finding is evident, prescribers frequently think broadly of 4 categories of pathogens among septic adults: gram-positive organisms (sometimes with special focus on *Enterococcus*), gram-negative organisms, anaerobes, and fungi. Anaerobes and fungi (mainly *Candida* spp) are relevant in certain syndromes (eg, intra-abdominal infections, catheter-related bloodstream infections) or other scenarios (eg, following a gastrointestinal endoscopic procedure); therefore, they should be managed based on a different set of prediction rules.^28^ The purpose of this calculator was to assist clinicians, at the bedside, with prescribing anti–gram-positive agents and anti–gram-negative empiric regimens.

Our study has several limitations. The tool is based on broadly applicable determinations, both in terms of the definitions we used for stratifying the level of resistance (ie, MDRO and XDRO), and our extrapolations to the spectrum of antibacterial coverage by agents. The calculator should also be validated in other centers, to determine its generalizability. On the validation data sets that it was tested, with all historical cohorts with data limited to specific subgroups, we observed a wide range of performances, some unfavorable. The performances with the validation data sets derive the overall performance of the tool. However, in 6 of the validation data sets, only patients with positive blood cultures were included, as opposed to the derivation data set, which included the entire background population from which the cases (ie, patients with MDRO or XDRO) arose. If MDROs or XDROs are not prevalent in a facility, the tool is irrelevant, though this situation is uncommon nowadays. In some hospitals, XDRO infections may be prevalent upon admission, but this situation is rare.^37^ The performance of the calculator might differ according to the percentages of MDROs and XDROs in different populations and might change over time, in accordance to temporal changes in MDROs and XDROs epidemiology. An additional limitation of our study is that the calculator does not address certain possible additional empiric prescribing dilemmas (eg, enterococcal, anaerobe, and fungal infections).^28^ However, it addresses the ‘backbone’ of empiric regimens for the majority of situations.

To conclude, we developed a tool to assist prescribers confronted with septic patients, which is free, anonymous, and accessible at the bedside, though it lacks additional validated data. Our future goal is to test the tool in a prospective interventional study on several outcomes, considering DAAT, mortality rates, and outcomes that measure the ecological burden of antimicrobials.

## Supplementary material

For supplementary material accompanying this paper visit https://doi.org/10.1017/ice.2020.1372. click here to view supplementary material
