# Supplementary material for: A “resistance calculator”: Simple stewardship intervention for refining empiric practices of antimicrobials in acute-care hospitals
Source: Infect Control Hosp Epidemiol. 2021 Mar 19;42(9):1082–9. doi: 10.1017/ice.2020.1372 (PMC8459314; doi:10.1017/ice.2020.1372)
Supplement: Supplementary file 1 [file S0899823X20013720sup001.docx]

**Supplementary Table 1- Characteristics of patients with sepsis upon admission or with nosocomial sepsis, Shamir (Assaf Harofeh) Medical Center, 2016.**

|  | | | | **Sepsis upon admission** | | **Nosocomial sepsis** | |
| --- | --- | --- | --- | --- | --- | --- | --- |
| **Parameter** | | | | **Frequency** | **Valid percent**^1^ | **Frequency** | **Valid percent** |
| Population (number of patients enrolled) | | | | 1,536 | | 802 | |
| **Demographics** | | | | | | | |
| Age (years) mean ± SD | | | | 62 ± 22 | | 77 (18-103) | |
| Female gender | | | | 869 | 56.6 | 367 | 45.8 |
| Elderly (> 65 years old) | | | | 827 | 53.8 | 618 | 77.1 |
| **Exposure to healthcare environments or procedures** | | | | | | | |
| Recent (<3 months) hospitalization | | | | 387 | 25.2 | 313 | 39 |
| LTCF resident | | | | 162 | 10.5 | 151 | 18.8 |
| Recent (<3 months) LTCF stay prior to hospitalization | | | | 490 | 31.9 | 176 | 21.9 |
| Regular visits (at least weekly) to outpatient clinics | | | | 79 | 5.1 | 120 | 15 |
| Hemodialysis | | | | 41 | 2.7 | 43 | 5.4 |
| Advanced nursing or intravenous therapy administered at home | | | | 23 | 1.5 |  |  |
| Antibiotic course (≥2 days duration) in the preceding 3 months | | | | 601 | 40.7 | 611 | 76.2 |
| Invasive procedure in the preceding 6 months | | | | 234 | 15.2 | 380 | 47.4 |
| Permanent device^1^ | | | | 173 | 11.3 | 187 | 23.3 |
| ICU stay in the preceding 3 months | | | | 13 | 0.8 | 23 | 8 |
| MDRO^2^ carrier from the preceding 2 years | | | | 183 | 11.9 | 265 | 33 |
| **Background medical status and conditions** | | | | | | | |
| Functionally dependent | | | | 540 | 35.2 |  |  |
| Chronic skin ulcers | | | |  |  | 212 | 26.4 |
| Altered consciousness / cognition | | | | 276 | 18 | 251 | 31.3 |
| Ischemic heart disease | | | | 323 | 21 | 288 | 35.9 |
| Congestive heart failure | | | | 208 | 13.5 | 318 | 39.7 |
| Diabetes mellitus | | | | 486 | 31.6 | 360 | 44.9 |
| Chronic kidney disease | | | | 249 | 16.2 | 205 | 25.6 |
| Chronic lung disease | | | | 277 | 18 | 297 | 37 |
| Active malignancy | | | | 142 | 9.2 | 164 | 20.4 |
| AIDS | | | | 3 | 0.2 | 1 | 0.1 |
| Immunosuppression^3^ | | | | 220 | 14.3 | 237 | 29.6 |
| Charlson's scores, mean ± standard deviation | | | Weighted Index Comorbidity | 2.1 ± 2.4 | | 5.2 ± 3.1 | |
|  |  |  | Combined Condition Score | 4.2 ± 3.5 | | 8± 3.8 | |
|  |  |  | 10 Year Survival, percent | 7.3 ± 6.8 | | 1 (0-98) | |
| **Acute illness indices** | | | | | | | |
| Severe sepsis, septic shock, multi-organ failure (previous sepsis severity classification) | | | | 444 | 28.9 | 319 | 39.8 |
| In ICU at culture date | | | | 121 | 7.9 | 321 | 40.1 |
| Mechanically ventilated | | | | 119 | 7.7 | 107 | 17.8 |
| Acute kidney injury | | | | 329 | 22.1 | 222 | 29.2 |
| Altered consciousness at culture date | | | | 380 | 24.7 | 438 | 54.6 |
| Rapidly fatal McCabe score | | | | 167 | 10.9 | 104 | 62.7 |
| Pitt score, median (IQR) | | | | 1 (0-1) | | 2 (0-14) | |
| Clinical syndrome | Urinary tract infection | | | 374 | 24.3 | 151 | 18.8 |
|  | Pneumonia and upper respiratory tract infection | | | 552 | 35.9 | 343 | 42.8 |
|  | Skin or soft tissue infection | | | 217 | 14.1 | 92 | 11.5 |
|  | Intra-abdominal infection | | | 185 | 12 | 50 | 6.2 |
|  | Endocarditis | | | 11 | 0.7 | 3 | 0.4 |
|  | Primary blood stream infection | | | 17 | 1.1 | 96 | 12 |
|  | Central nervous system infection | | | 22 | 1.4 | 2 | 0.2 |
|  | Bacteremia without determined focus | | | 39 | 2.5 | 64 | 8 |
|  | Gynecologic / pelvic infection | | | 119 | 7.7 | 1 | 0.1 |
| Hospitalization division | Medicine (including advanced-care rooms) | | | 976 | 63.5 | 327 | 41 |
|  | Surgery | | | 157 | 10.2 | 148 | 18 |
|  | Obstetrics-Gynecology | | | 201 | 13.1 | 8 | 1 |
|  | ICU or ICCU | | | 132 | 8.6 | 319 | 40 |
|  | Emergency Room | | | 70 | 4.6 | NA | NA |
| **Antimicrobial therapy** | | | | | | | |
| Days to initiation of appropriate therapy, median (range) | | | | 0 (0-2) | | 1 (0-12) | |
| ≥ 48 hours delay in initiating appropriate therapy | | | | 130 | 26.4 | 63 | 22.2 |
| **Outcomes** | | | | | | | |
| Length of stay from infection to discharge, after excluding the dead, days, median (IQR) | | | | 5 (3-8) | | 22 (5-669) | |
| Died during current hospitalization | | | | 142 | 9.2 | 287 | 35.8 |
| Died during 14 days after culture date | | | | 105 | 7 | 223 | 27.8 |
| Died during 90 days after culture date | | | | 217 | 16.5 | 363 | 45.3 |
| Among survivors of index hospitalization | | Functional status deterioration at discharge | | 140 | 10 | 255 | 50 |
|  |  | Discharge to LTCF (only patients admitted from home) | | 60 | 4.7 | 176 | 39 |
|  |  | *Clostridium difficile* infection in 90 days | | 7 | 0.9 |  |  |
|  |  | Additional hospitalization in the following 3 months | | 348 | 29.6 | 158 | 35.6 |

^1^ data is presented as valid percent- unknown data was removed from statistics.

^2^ MDRO- multi-drug resistant organisms included pathogens that were resistant to the first empiric antibiotic line: Methicillin-resistant *Staphylococcus aureus* (MRSA); Ampicillin and/or vancomycin resistant Enterococcus; Penicillin and/or ceftriaxone non-susceptible *Streptococcus pneumoniae*; *Acinetobacter baumannii*; *Pseudomonas aeruginosa*; Enterobacteriaceae non-susceptible to ≥1 3^rd^ generation cephalosporin (e.g., ceftriaxone, ceftazidime, cefotaxime); *Stenotrophomonas maltophilia*.

^3^ Immunosuppression include either of the following situations: neutropenia (<500 neutrophils) present at day of culture; Glucocorticoid use for >48 hours in the month previous to culture day; chemotherapy or radiotherapy in the 3 months previous to culture day; carrier of Human Immunodeficiency Virus; Patient has had a bone marrow or solid organ transplantation; Anti-Tumor Necrosis Factor of Anti-tyrosine kinase therapy in past 3 months

SD – standard deviation; LTFC – Long Term Care Facility; S/P – stats post; CABG – coronary artery bypass graft; CVA - cerebrovascular accident; TIA - Transient Ischemic Attack; AIDS - Acquired Immune Deficiency Syndrome; SIRS - Systemic Inflammatory Response Syndrome; ICU - intensive care unit; ICCU - Intensive Cardiac-Care Unit. Ob/Gyn- Obstetrics or Gynecology; IQR – inter quartile range.

**Supplementary Table 2 – Characteristics and features of the validation datasets included in the study**.

| Validation datasets of the MDR upon admission score | | | | | Validation datasets of the nosocomial XDR score | | | | |
| --- | --- | --- | --- | --- | --- | --- | --- | --- | --- |
| Dataset name | PI | Pt. No. | Period | Description | Dataset name | PI | Pt. No. | Period | Description |
| SMC MDR1 | Gil Marcus | 426 | 1/12/2014 – 4/25/2014 | SMC, patients with MDR BSI vs. patients with non-MDR BSI | DMC CRE1 | Violet Libman | 182 | 2007-2010 | DMC, patients with CRE BSI vs. patients with ESBL BSI |
| SMC MDR2 | Mor Broide | 199 | 7/1/2012 - 6/30/2013 | SMC, patients with ESBL-producing Enterobacterales infections vs. patients with non-ESBL-producing Enterobacterales infections | SMC XDR1 | Matar Yekutiel | 257 | 1/1/2013- 2/28/2013 | SMC, patients with XDR BSI vs. patients with non-XDR BSI |
| SMC MDR3 | Eyal Taleb | 311 | 1/1/2013- 7/31/2013 | SMC, patients with MDR infections vs. patients with non-MDR infections | SMC XDR2 | Dana Leveat | 162 | 1/1/2013- 7/31/2013 | SMC, patients with nosocomial BSI vs. patients with community-onset (i.e., both community-acquired and healthcare-associated) BSI |
|  |  |  |  |  | SMC XDR3 | Itzhak Vitkon-Barkay | 239 | 1/1/2007- 5/31/2012 | SMC, patients with XDR infections vs. patients with MDR infections |

PI= primary investigator; Pt= patient; No= number; MDR= multi-drug resistant; XDR= extensively drug resistant; SMC= Shamir Medical Center; BSI= bloodstream infection; DMC= Detroit Medical Center; CRE= carbapenem-resistant Enterobacterales; ESBL= extended-spectrum beta-lactamase producing Enterobacterales.

**Supplementary Table 3 - Frequencies of pathogens in the developing cohorts, Shamir (Assaf Harofeh) Medical Center, 2016**.

|  | | | | **MDRO upon-admission** | | | **Nosocomial XDRO** | | |
| --- | --- | --- | --- | --- | --- | --- | --- | --- | --- |
|  | | | | **Frequency** | **Valid percent^1^** | **Frequency** | | **Valid percent^1^** |  |
| **Blood bacteria isolation** | | | | | | | | | |
| Bacteremia | | | | 19.3 | 296 | 201 | | 40 |  |
| Polymicrobial isolations | | | | 11.7 | 35 | 176 | | 35 |  |
| **Representative organisms’ group/type** | | | | | | | | | |
| Aerobic Gram positive bacteria | | | | 148 | 29.5 | 78 | | 15.5 |  |
| Aerobic Gram negative bacteria | | | | 351 | 70.1 | 370 | | 73.5 |  |
| **Organism type** | | **Organism's full name** | | **Frequency** | **Valid percent^1^** | **Frequency** | | **Valid percent^1^** |  |
| Aerobic GPC | | *Enterococcus* spp. | | 27 | 5.4 | 35 | | 7.8 |  |
|  |  | *Staphylococcus aureus* | | 53 | 10.6 | 36 | | 8 |  |
|  |  | Coagulase negative staphylococci | | 66 | 13.2 | 6 | | 1.3 |  |
|  |  | *Lactobacillus* spp. | | 1 | 0.2 | 0 | |  |  |
|  |  | *Listeria monocytogenes* | | 1 | 0.2 | 0 | |  |  |
|  |  | *Corynebacterium* spp. | | 1 | 0.2 | 0 | |  |  |
|  |  | Viridans streptococci | | 62 | 12.4 | 1 | | 0.2 |  |
| Aerobic GNB | | *Achromobacter xylosoxidans* | | 2 | 0.4 | 2 | | 0.4 |  |
|  |  | *Acinetobacter baumannii* | | 7 | 1.4 | 108 | | 24 |  |
|  |  | *Acinetobacter haemolyticus* | | 1 | 0.2 |  | |  |  |
|  |  | *Acinetobacter junii* | | 0 |  | 1 | | 0.2 |  |
|  |  | *Acinetobacter lwoffii* | | 0 |  | 2 | | 0.4 |  |
|  |  | *Burkholderia cepacia* | | 0 |  | 1 | | 0.2 |  |
|  |  | *Salmonella* spp. | | 4 | 0.8 |  | |  |  |
|  |  | *Citrobacter* spp. | | 7 | 1.4 | 3 | | 0.6 |  |
|  |  | *Enterobacter* spp. | | 11 | 2.2 | 20 | | 4.4 |  |
|  |  | *Moraxella catarrhalis* | | 4 | 0.8 | 0 | |  |  |
|  |  | *Mycoplasma hominis* | | 1 | 0.2 | 0 | |  |  |
|  |  | *Escherichia coli* | | 152 | 30.3 | 50 | | 11.2 |  |
|  |  | *Haemophilus influenzae* | | 8 | 1.6 | 3 | | 0.7 |  |
|  |  | *Klebsiella pneumoniae* | | 59 | 11.8 | 58 | | 12.9 |  |
|  |  | *Morganella morganii* | | 6 | 1.2 | 5 | | 1.1 |  |
|  |  | *Pantoea agglomerans* | | 1 | 0.2 | 1 | | 0.2 |  |
|  |  | *Proteus mirabilis* | | 16 | 3.1 | 24 | | 5.3 |  |
|  |  | *Proteus penneri* | |  |  | 1 | | 0.2 |  |
|  |  | *Providencia stuartii* | | 3 | 0.6 | 4 | | 0.9 |  |
|  |  | *Pseudomonas aeruginosa* | | 40 | 8 | 57 | | 12.7 |  |
|  |  | Other *Pseudomonas* spp. | | 5 | 1 |  | |  |  |
|  |  | *Serratia* spp | | 4 | 0.8 | 4 | | 0.9 |  |
|  |  | *Shigella* spp. | | 7 | 1.4 | 0 | |  |  |
|  |  | *Sphingomonas paucimobilis* | | 2 | 0.4 | 1 | | 0.2 |  |
|  |  | *Stenotrophomonas maltophilia* | | 7 | 1.4 | 25 | | 5.6 |  |
| Mycobacteria | | *Mycobacterium tuberculosis* | | 2 | 0.4 | 0 | |  |  |
| **MDRO** | | | | | | | | | |
| Gram positives | *Staphylococcus aureus* | | | 12 | 7.3 |  | |  |  |
|  | *Enterococcus faecalis* | | | 2 | 1.2 |  | |  |  |
|  | *Enterococcus faecium* | | | 2 | 1.2 |  | |  |  |
| Gram negatives | *Escherichia coli* | | | 45 | 27.3 |  | |  |  |
|  | *Pseudomonas aeruginosa* | | | 41 | 24.8 |  | |  |  |
|  | *Klebsiella pneumoniae* | | | 28 | 17 |  | |  |  |
|  | *Proteus mirabilis* | | | 10 | 6.1 |  | |  |  |
|  | *Acinetobacter baumannii* | | | 7 | 4.2 |  | |  |  |
|  | *Stenotrophomonas maltophilia* | | | 7 | 4.2 |  | |  |  |
|  | *Morganella morganii* | | | 3 | 1.8 |  | |  |  |
|  | *Providencia stuartii* | | | 3 | 1.8 |  | |  |  |
|  | *Achromobacter xylosoxidans* | | | 1 | 0.6 |  | |  |  |
|  | *Enterobacter aerogenes* | | | 1 | 0.6 |  | |  |  |
|  | *Enterobacter asburiae* | | | 1 | 0.6 |  | |  |  |
|  | *Pseudomonas alcaligenes* | | | 1 | 0.6 |  | |  |  |
|  | *Sphingomonas paucimobilis* | | | 1 | 0.6 |  | |  |  |
| **XDRO group/type** | | | | | | | | | |
| GPC | | | |  |  | 2 | | 1.2 |  |
| GNB | | | |  |  | 165 | | 98.8 |  |
| **XDRO type** | | **XDRO's full name** | |  |  |  | |  |  |
| Aerobic GPC | | VRE | |  |  | 2 | | 0.2 |  |
| Aerobic GNB | | *Achromobacter xylosoxidans* | |  |  | 1 | | 0.1 |  |
|  |  | CRAB | |  |  | 100 | | 12.5 |  |
|  |  | *Burkholderia cepacia* | |  |  | 1 | | 0.1 |  |
|  |  | CRE | *Enterobacter aerogenes* |  |  | 1 | | 0.1 |  |
|  |  |  | *Enterobacter cloacae* |  |  | 1 | | 0.1 |  |
|  |  |  | *Escherichia coli* |  |  | 1 | | 0.1 |  |
|  |  |  | *Klebsiella pneumoniae* |  |  | 16 | | 2.1 |  |
|  |  | CRPA | |  |  | 19 | | 2.4 |  |
|  |  | *Stenotrophomonas maltophilia* | |  |  | 25 | | 3.1 |  |

^1^ Valid percent represents the frequency percent after exclusion of items for which data was missing (those were subtracted from the denominator).

MDRO- multi-drug resistance organisms; XDRO- extensively drug-resistant organisms; CRAB – carbapenem-resistant *Acinetobacter baumannii*; CRE – carbapenem -resistant enterobacteriaceæ; CRPA – carbapenem-resistant *Pseudomonas aeruginosa*; GNB – Gram-negative bacilli; VRE- vancomycin-resistant *Enterococcus*; GPC- Gram positive cocci.
